# Evaluating the impact of Carbon Emission Trading Policy on pan-cancer incidence among middle-aged and elderly populations: a quasi-natural experiment

**DOI:** 10.1265/ehpm.24-00387

**Published:** 2025-05-29

**Authors:** Chuang Yang, Yiyuan Sun, Yihan Li, Lijun Qian

**Affiliations:** 1 The First Affiliated Hospital with Nanjing Medical University, Nanjing, China; 2 Business School, University of International Business and Economics, Beijing, China

**Keywords:** Carbon Emission Trading Policy, Cancer incidence, Environmental pollution, Psychological health, Public health

## Abstract

**Background:**

Cancer is a major public health concern, particularly among middle-aged and elderly populations, who are disproportionately affected by rising cancer incidence. Environmental pollution has been identified as a significant risk factor for cancer development. China’s Carbon Emission Trading Policy (CETP), implemented in pilot regions since 2013, aims to reduce carbon emissions and improve air quality. This study evaluates the impact of CETP on pan-cancer incidence, with a focus on its effects on specific cancer types and vulnerable populations.

**Methods:**

This quasi-natural experiment utilized data from the China Health and Retirement Longitudinal Study (CHARLS) and environmental data from the China National Environmental Monitoring Center (2011–2018). A staggered difference-in-differences (DID) model was employed to estimate the impact of CETP on cancer incidence. Robustness tests, including parallel trend tests, placebo analysis, and entropy balancing, validated the findings. Subgroup analyses were performed to assess the policy’s heterogeneous effects based on gender, Body Mass Index (BMI), and smoking status.

**Results:**

CETP implementation significantly reduced the incidence of six cancer types: endometrial, cervical, gastric, esophageal, breast, and lung cancers. Overall, pan-cancer incidence significantly declined post-policy implementation (CETP × POST: −47.200, 95% CI: [−61.103, −33.296], p < 0.001). The policy demonstrated stronger effects in highly polluted areas and among individuals with poorer mental health. Subgroup analysis revealed that females, individuals with lower BMI, and non-smokers experienced more substantial benefits.

**Conclusions:**

CETP significantly reduces cancer incidence by improving environmental quality and influencing mental health, with particularly strong effects observed among high-risk populations. This study highlights the important role of environmental economic policies in mitigating cancer burden and promoting public health. Future research should further explore the long-term impacts of this policy and its applicability across different national and regional contexts.

**Supplementary information:**

The online version contains supplementary material available at https://doi.org/10.1265/ehpm.24-00387.

## Introduction

Cancer is a growing global public health issue, especially among middle-aged and elderly populations, where incidence and mortality rates are continuously rising, imposing a heavy burden on society and families [1–3]. In China, approximately 24% of the world’s new cancer cases occur annually, making cancer a major threat to the health of middle-aged and elderly people [4, 5]. This group is particularly prone to multiple types of cancer, such as lung, gastric, liver, and breast cancers, with incidence rates significantly increasing with age. At the same time, accumulating evidence suggests that the development of several cancers, including breast, cervical, and endometrial cancers, is also closely linked to psychosocial factors (e.g., stress, mental health) and lifestyle behaviors (e.g., diet, smoking status), alongside environmental exposures [6–9]. Consequently, reducing cancer incidence and improving cancer prevention have become urgent public health challenges.

Environmental pollution has been widely recognized as a key factor influencing cancer development [10, 11]. Numerous studies have demonstrated a significant association between air pollutants (e.g., PM2.5, NO_2_, SO_2_) and the risk of various cancers, especially in highly urbanized and industrialized areas [12–14]. Long-term exposure to air pollution can notably increase the risk of lung cancer, which is often considered one of the most sensitive malignancies to particulate matter and other pollutants [11–13]. However, hormone-related cancers—including breast, cervical, and endometrial cancers—can also be affected by environmental contaminants and may be further exacerbated by internal psychological factors and lifestyle-related risks. Thus, improving environmental quality is viewed as a critical means to mitigate a broad range of cancer risks, yet many knowledge gaps remain regarding how different cancers may respond to pollution-reduction policies under varying socioeconomic and psychosocial contexts.

To address the global health risks associated with environmental pollution, various countries have implemented carbon reduction policies. From 2013 to 2020, the Chinese government gradually implemented the Carbon Emission Trading Policy (CETP) in eight pilot regions, including Shenzhen, aiming to control carbon emissions and improve air quality through market-based mechanisms [15, 16]. This policy, which sets carbon emission quotas and encourages enterprises to reduce emissions, has the potential not only to mitigate climate change but also to reduce pollution and improve public health [15]. Importantly, CETP may yield broader impacts beyond environmental quality alone: as an economic intervention, it can also reshape regional industries and influence residents’ living standards, behaviors, and mental well-being [16, 17]. These secondary effects could, in turn, modify cancer risk through stress reduction, improved healthcare access, and shifts in lifestyle choices such as smoking or diet. Despite these promising avenues, there is still a lack of systematic empirical research on the specific impact of CETP on multiple cancer types, particularly among middle-aged and elderly populations in highly polluted areas. To fill this gap, the present study focuses on six cancers—lung, breast, esophageal, gastric, cervical, and endometrial, which represent a significant burden of morbidity among Chinese adults, and are influenced by overlapping yet distinct factors, including environmental pollution, psychosocial stress, and behavioral risks. By centering on these cancers, we aim to explore whether and how CETP implementation is associated with changes in cancer incidence and whether this effect varies across cancers with potentially diverse etiological pathways.

## Methods

### Data sources and study design

This study used a comprehensive dataset that combines health and environmental data to investigate the impact of CETP on pan-cancer incidence among middle-aged and elderly populations in China. The primary health data comes from the China Health and Retirement Longitudinal Study (CHARLS), a nationally representative longitudinal survey targeting Chinese residents aged 45 and above [18]. During data cleaning, missing values were addressed by excluding participants with incomplete key covariates, and outliers were carefully reviewed and removed to ensure data integrity and reliability. CHARLS initially recruited 17,705 participants in 2011 as the baseline survey. Follow-up surveys were conducted biennially, each wave including additional participants. From 2011 to 2018, four waves of follow-up were completed, covering 77,223 participants (17,705 in 2011; 18,606 in 2013; 21,096 in 2015; and 19,816 in 2018). From this cohort, individuals younger than 45 years or those who moved during follow-up (n = 2,388) were excluded, as well as those with a pre-existing cancer diagnosis at the 2011 baseline (n = 603). Due to missing data on relevant covariates, an additional 36,183 participants were excluded, resulting in a final sample of 41,040 participants (Fig. S1). Written informed consent was obtained from all participants or their legal representatives before baseline and follow-up surveys.

Environmental quality data were obtained from the China National Environmental Monitoring Center, including pollutants such as ozone (O_3_), nitrogen dioxide (NO_2_), carbon monoxide (CO), sulfur dioxide (SO_2_), PM2.5, and PM10. These data were mainly used to analyze the association between environmental pollution and cancer incidence among elderly populations.

The “China Urban Statistical Yearbook” provided data on industrial sulfur dioxide, industrial dust, and industrial wastewater emissions. We also included annual average PM2.5 concentration data for Chinese cities from 2011 to 2018, obtained from the Atmospheric Composition Analysis Group at Dalhousie University. We used the entropy weight method to calculate the Environmental Pollution Index to assess the moderating effect of environmental pollution between CETP and cancer incidence [19]. To ensure precise matching across different datasets, the participants’ permanent residence was used as the unique identifier for linking data.

### Outcome and covariates

The primary outcome of this study was the occurrence of cancer events, assessed through standardized questionnaires. Participants were asked in the “Health Status and Functioning” section: “Have you been diagnosed with cancer or a malignant tumor by a doctor (excluding minor skin cancers)?” If the answer was “yes,” they were further asked: “In which organ or part of your body do you have cancer?” including both the primary site and metastatic sites. Options included the following organs or body parts: brain/mouth/larynx/other pharynx/thyroid/lung/breast/esophagus/stomach/liver/pancreas/kidney/prostate/testicle/ovary/cervix/endometrium/colon or rectum/bladder/skin/non-Hodgkin lymphoma/leukemia/other organs.

Covariate data were collected from CHARLS based on biological or clinically relevant factors, including demographic information, health status, sample information [20]. Covariates included gender (male, female), age (years), education level (below high school, high school and above), residence (urban, rural), alcohol consumption, smoking status, body mass index (BMI, kg/m^2^), sleep duration (hours), mental health score (measured by the short version of the Center for Epidemiologic Studies Depression Scale (CESD)) [21], hypertension history, and diabetes history.

### Statistical analysis

The Chinese CETP started in 2013 as a regional pilot project, with Shenzhen, Beijing, Shanghai, Guangdong, and Tianjin being the first to conduct carbon trading. Chongqing and Hubei joined in 2014, followed by Fujian in 2016 [22]. We used the CETP pilot as an exogenous shock to establish a quasi-natural experiment, employing a staggered difference-in-differences (DID) model to observe pan-cancer risk among participants before and after policy implementation in pilot and non-pilot regions.

The DID model is represented as follows:

$$\begin{array}{rl} {\text{Cancer}}_{it} & =\alpha_{0}+\alpha_{1}{\text{treat}}_{i}\times {\text{post}}_{it}+\lambda X_{it}\\ & \;+\text{Fixed Effect}+\varepsilon_{it} \end{array}$$ (1)

In model (1), Subscript i denotes the individual participant, and t represents time. The term *treat_i_* × *post_it_* is the core explanatory variable of interest, indicating whether the participant’s region was included in the CETP pilot. If the region was included in the pilot, *treat_i_* × *post_it_* takes the value of 1 for that year and subsequent years; otherwise, it is 0. *X_it_* represents individual-level control variables. *Fixed Effect* represents time-fixed effects controlled for in the model, and ε_it_ denotes the random error term.

Before applying the staggered DID model, we conducted a parallel trend test as follows:

$$\begin{array}{rl} {\text{cancer}}_{i,t} & =\beta_{0}+\sum_{i=-1}^{-1}\beta_{l}^{0}D_{i,t}^{l}+\sum_{i=0}^{3}\beta_{l}^{1}D_{i,t}^{l}\\ & \;+{\text{Controls}}_{i,t}+\text{Fixed Effect}+\varepsilon_{i,t} \end{array}$$ (2)

In model (2), $D_{i,t}^{l}$ represents dummy variables indicating whether an individual was included in the policy pilot at each time point (1 if in a pilot region, otherwise 0). The third phase was excluded as the reference group to avoid potential collinearity. To intuitively determine the parallel trend assumption of the DID method, we plotted the β coefficients and their 95% confidence intervals for the two years before and three years after the implementation of carbon trading. *Fixed Effect* represents time-fixed effects controlled for in the model, and ε_it_ denotes the random error term.

To ensure the robustness of the results, we also used a non-parametric permutation test for a placebo test, where participants from pilot regions were randomly assigned, as well as the timing of the carbon emissions trading policy implementation. This process was repeated 500 times. Additionally, we employed Propensity Score Matching (PSM) and Entropy Balancing. Entropy Balancing adjusts weights to match the covariate distributions between the treatment and control groups. This method uses an optimization algorithm to minimize the discrepancy between the reweighted and the original data, thereby ensuring that all covariates are balanced across groups. Compared with PSM, Entropy Balancing offers the advantage of precisely balancing the distribution of all covariates without requiring explicit matching pairs. It also avoids sample loss, providing more stable and accurate estimates.

To explore the moderating role of internal and external factors in the policy’s impact on pan-cancer incidence, we constructed the following model:

$$\begin{array}{rl} {\text{Cancer}}_{it} & =\alpha_{0}+\alpha_{1}{\text{Modulator}}_{it}+\alpha_{2}{\text{treat}}_{i}\times {\text{post}}_{it}\\ & \;+\alpha_{3}{\text{treat}}_{i}\times {\text{post}}_{it}\times {\text{Modulator}}_{it}+\lambda X_{it}\\ & \;+\text{Fixed Effect}+\varepsilon_{it} \end{array}$$ (3)

In model (3), Subscript i denotes the individual participant, and t represents time. The term *treat_i_* × *post_it_* is the core explanatory variable of interest, indicating whether the participant’s region was included in the CETP pilot. If the region was included in the pilot, *treat_i_* × *post_it_* takes the value of 1 for that year and subsequent years; otherwise, it is 0. *Modulator_it_* refers to either external environmental pollution index or internal psychological health scores used to validate moderating effects. *X_it_* represents individual-level control variables. *Fixed Effect* represents time-fixed effects controlled for in the model, and ε_it_ denotes the random error term.

All statistical analyses were conducted using Stata 18.0 and R (v4.1.1). Statistical significance was determined by a two-tailed p-value of <0.05.

## Results

### 1. Impact of CETP on pan-cancer incidence

**Fig. 1:**
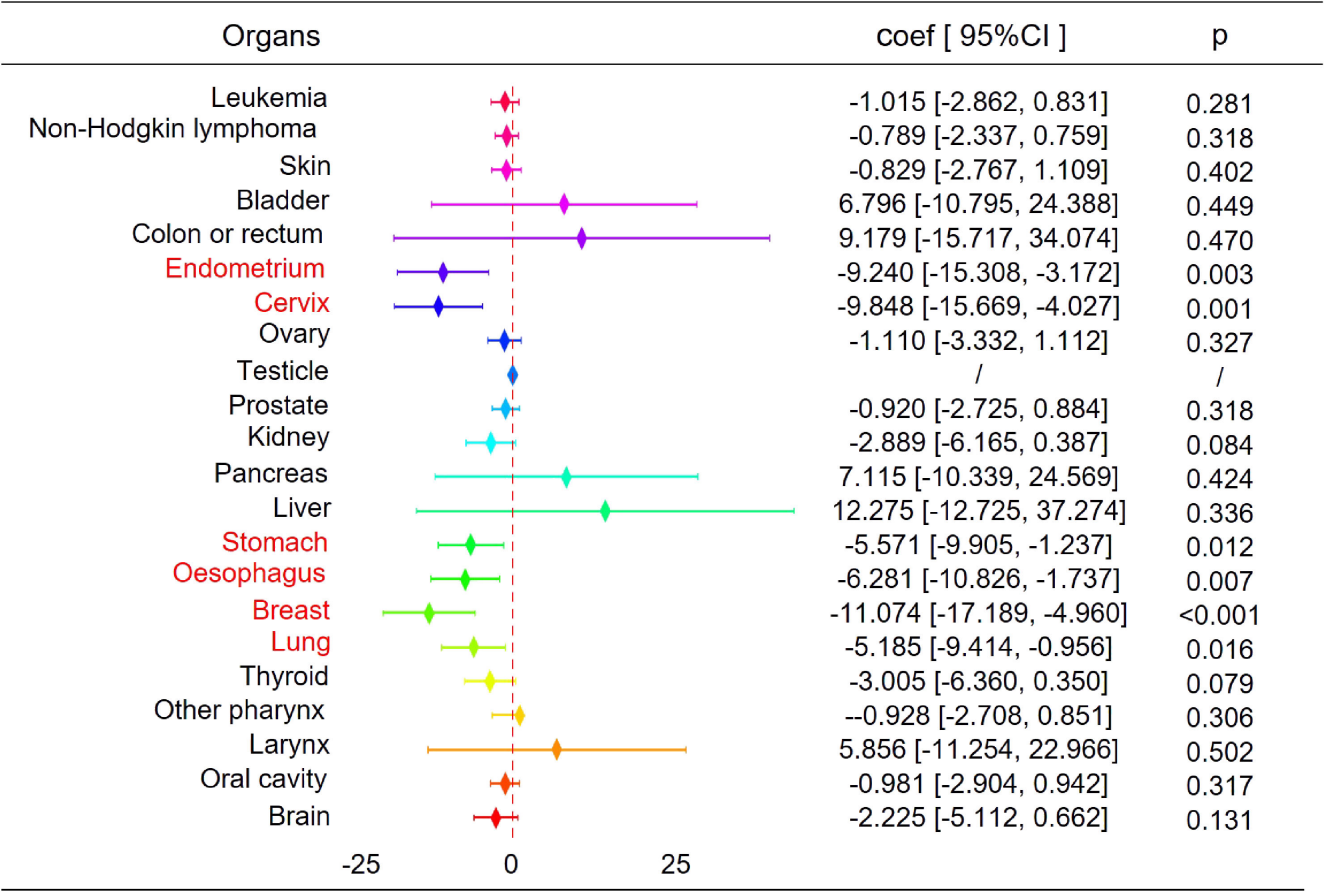
Impact of CETP on Incidence of Different Cancer Types

Prior to applying the staggered DID model, we conducted a parallel trend test to evaluate its applicability [23]. Based on the results of Table S1 and Fig. S2, the coefficients of the CETP implementation dummy variable are not statistically significant during the pre-policy and implementation periods, while the coefficients become significantly negative at the 1% level in the two post-implementation periods. This finding validates the parallel trend assumption and supports the use of the DID model for causal inference. Figure 1 shows the impact of CETP on the incidence of 22 different cancers. CETP significantly reduced the incidence of six types of cancer, including endometrial cancer, cervical cancer, gastric cancer, esophageal cancer, breast cancer, and lung cancer. For example, the regression coefficients for cervical cancer and breast cancer were −9.848 (95% CI: [−15.669, −4.027], p = 0.001) and −11.074 (95% CI: [−17.189, −4.960], p < 0.001), respectively. These results suggest that CETP implementation significantly reduced the incidence of these cancers, while the impact on some other cancer types was not significant or showed no clear trend.

To further evaluate CETP’s overall effect, we combined the six cancer types with significant effects into a pan-cancer index and conducted an interaction analysis (Table 1). In Model 1, the coefficient for CETP × POST was −41.060 (95% CI: [−59.880, −22.240], p < 0.001), indicating a significant decrease in pan-cancer incidence after policy implementation. Gender and age also significantly affected pan-cancer risk, with a coefficient for females of −42.241 (95% CI: [−54.368, −30.114], p < 0.001). In Model 2, which included additional covariates, the coefficient for CETP × POST was −47.200 (95% CI: [−61.103, −33.296], p < 0.001), indicating that the effect of the policy remained robust even after controlling for various confounding factors. Lifestyle factors, such as sleep duration and smoking status, were also significantly associated with cancer risk, further emphasizing the importance of considering individual health behaviors in cancer prevention strategies.

**Table 1:** Impact of CETP on Pan-cancer Incidence^#^

**Variables**	**Model 1** **Coef [95% CI]**	**p**	**Model 2** **Coef [95% CI]**	**p**
CETP × POST	−41.060 [−59.880, −22.240]	<0.001	−47.200 [−61.103, −33.296]	<0.001
Gender	−42.241 [−54.368, −30.114]	<0.001	−26.252 [−44.794, −7.710]	0.006
Age	−0.715 [−1.406, −0.023]	0.043	−0.592 [−1.451, 0.267]	0.177
BMI	0.011 [−2.177, 2.200]	0.992	0.187 [−2.484, 2.858]	0.891
Education			4.091 [−3.453, 11.635]	0.288
Rural			−8.297 [−23.227, 6.632]	0.276
Sleep			−4.579 [−8.214, −0.945]	0.014
Smoke			−17.569 [−31.169, −3.969]	0.011
Drink			−9.519 [−25.964, 6.925]	0.257
Hypertension			−1.515 [−16.780, 13.749]	0.846
Diabetes			−1.048 [−21.639, 19.543]	0.921
_cons	102.739 [29.854, 175.625]	0.006	117.916 [29.371, 206.460]	0.009
r2	0.001		0.001	
N	41040		34399	

^#^Model 1 controls for core variables including gender, age, and BMI. Model 2 further incorporates additional covariates, such as education, rural residency, sleep duration, smoking status, alcohol consumption, hypertension history, and diabetes history.

### 2. Robustness tests for CETP effect on pan-cancer

In empirical analyses involving DID, there may be bias in standard errors due to serial correlation, which can lead to over-rejection of the null hypothesis [24]. To ensure the robustness of the study results, we first conducted a placebo test using a non-parametric permutation approach [25, 26]. Figure S3 shows the probability distribution and kernel density distribution of placebo variables during random sampling, revealing that regression coefficients are centered around 0, indicating a normal distribution, and the baseline regression estimate (−0.005) is an unlikely event within the probability distribution of the placebo policy variable. Thus, the reduction in cancer risk following CETP implementation is not due to chance.

In addition, to mitigate potential biases caused by individual-level differences, we further applied Entropy Balancing and PSM to validate the robustness of the findings [29]. Regression analyses conducted on the datasets processed through entropy balancing and PSM show that CETP has significant negative effects on the incidence of lung cancer, breast cancer, esophageal cancer, gastric cancer, cervical cancer, and endometrial cancer (Table S2). To address potential sample selection bias resulting from missing data, we employed an imputation approach for BMI and confirm that our main conclusions remain robust (Table S3) [27]. Furthermore, as a robustness test, we re-included newly migrated individuals, defined as those who entered the sample frame after the baseline survey. The results, presented in Table S4, are consistent with our primary findings, providing further support for the robustness of our conclusions.

Given that the implementation of CETP overlaps with several major environmental policies launched around the same time in China, we included relevant initiatives such as the Low-Carbon City Pilot Program and the Energy Use Rights Trading Pilot Program as control variables to eliminate potential interference. After controlling for these other factors, the effect of carbon emissions trading remains significant, indicating that the policy impact can be more credibly attributed to CETP itself. We also added years of residence as a control variable in the regression model and the results remain robust, providing further support for the validity of our conclusions (Table S5).

### 3. Effect of CETP and the moderating role of external and internal factors

We used the environmental pollution index and psychological health scores as external and internal moderating factors to assess whether CETP’s effectiveness in reducing cancer incidence was moderated by these factors (Table 2). Firstly, the correlation between common air pollutants (e.g., O_3_, NO_2_, CO, SO_2_, PM2.5, PM10) and various cancers is well-known [14, 28–31]. Figure S4 shows significant correlations between specific pollutants and certain types of cancer, indicating that air pollution is a major risk factor for cancers such as breast and cervical cancers, consistent with existing literature linking environmental pollutants to cancer risk. Furthermore, the environmental pollution index had a significantly positive impact on cancer incidence (p = 0.011), indicating that higher levels of pollution could increase cancer risk. However, after CETP implementation, the interaction between pollution index and policy showed a significant negative relationship (p = 0.011), suggesting that the policy significantly reduced cancer incidence in highly polluted areas, reflecting its positive role in mitigating the adverse health effects of pollution.

**Table 2:** Moderating Effects of Pollution Index and Psychological Health on CETP Impact on Pan-Cancer Incidence

**Variables**	**Pan-cancer**			

	**coef 1** **[95% CI]**	**p**	**coef 2** **[95% CI]**	**p**
CETP × Post	−39.661 [−56.001, −23.321]	<0.001	−20.740 [−37.977, −3.502]	0.018
Pollution Index	1161.046 [269.913, 2052.179]	0.011		
Pollution Index × CETP × Post	−1165.341 [−2062.712, −267.970]	0.011		
CES-D-10			3.045 [1.456, 4.634]	<0.001
CES-D-10× CETP × Post			−3.248 [−4.710, −1.786]	<0.001
_cons	121.106 [29.622, 212.590]	0.009	66.383 [−23.600, 156.367]	0.148
r2	0.002		0.002	
N	30726		30726	

Moreover, psychological health scores were significantly positively associated with cancer incidence (p < 0.001), indicating that poor mental health was linked to an increased risk of cancer. However, the interaction between psychological health scores and CETP implementation yielded a coefficient of −3.248, p < 0.001, suggesting that the policy significantly reduced cancer incidence in individuals with poorer mental health, highlighting its beneficial effect on vulnerable populations.

### 4. Subgroup analysis

To further assess the differential impact of CETP on various populations, we considered factors such as gender, BMI, and smoking status for subgroup analysis (Table 3). Results showed that CETP significantly reduced cancer incidence in both males and females, with the effect being more pronounced in females (coefficient = −64.741, 95% CI: [−87.189, −42.294], p < 0.001). In contrast, the coefficient for males was −30.216 (95% CI: [−46.055, −14.377], p < 0.001), indicating that the reduction in cancer incidence due to CETP was more notable in females. For BMI, cancer incidence significantly decreased among individuals with underweight BMI (<18.5), with a coefficient of −97.993 (95% CI: [−173.951, −22.036], p = 0.011). Among those with normal BMI (18.5 to 25), cancer incidence also significantly decreased (coefficient = −40.798, 95% CI: [−57.176, −24.421], p < 0.001). However, no significant decrease in cancer incidence was observed among individuals with BMI ≥ 25 (coefficient = −7.496, 95% CI: [−27.520, 12.528], p = 0.463), indicating a weaker response to the policy among individuals with higher BMI. Additionally, smoking status significantly influenced CETP’s effect. Cancer incidence significantly decreased among non-smokers (coefficient = −63.106, 95% CI: [−81.879, −44.334], p < 0.001), while no significant effect of the policy was observed among smokers (coefficient = −13.451, 95% CI: [−27.727, 0.825], p = 0.065). Chow tests between each subgroup (p < 0.001) indicated significant differences between different subgroups, suggesting that CETP’s effectiveness in reducing cancer incidence varied based on gender, BMI, and smoking status.

**Table 3:** Subgroup Analysis* of CETP Impact on Pan-Cancer Incidence

	**coef [95% CI]**	**p**
Gender		
male	−30.216 [−46.055, −14.377]	<0.001
female	−64.741 [−87.189, −42.294]	<0.001
BMI		
<18.5	−97.993 [−173.951, −22.036]	0.011
18.5–25	−40.798 [−57.176, −24.421]	<0.001
>=25	−7.496 [−27.520, 12.528]	0.463
Smoke		
No	−63.106 [−81.879, −44.334]	<0.001
Yes	−13.451 [−27.727, 0.825]	0.065

*Chow test p < 0.001

## Discussion

We systematically evaluated the impact of CETP as an innovative intervention on the incidence of multiple cancers. Our study demonstrated that CETP significantly reduced the incidence of several cancers, including endometrial, cervical, gastric, esophageal, breast, and lung cancers. Pan-cancer incidence decreased significantly after policy implementation, and the effect remained robust even after adjusting for gender, age, lifestyle factors, and other confounders. The parallel trend test and placebo test further validated the validity and robustness of the model. By incorporating Entropy Balancing and PSM to adjust for individual differences and including internal and external moderating factors such as environmental pollution and mental health, the rigor of the results was further enhanced. In terms of moderating roles, the environmental pollution index was positively associated with cancer incidence, but after policy implementation, the interaction between pollution and policy was negative, indicating a significant reduction in cancer risk in highly polluted areas. Similarly, poor mental health was associated with higher cancer risk, but CETP implementation significantly reduced cancer incidence in those with poorer mental health. Subgroup analyses highlighted that females, those with lower BMI, and non-smokers benefited more from the policy. This multi-level analysis not only revealed the overall positive impact of CETP on cancer incidence but also demonstrated the policy’s significant effects in specific populations, providing new insights and empirical support for the effectiveness of environmental policies in public health.

Numerous studies have shown a significant relationship between air pollution and various cancers (e.g., lung and breast cancers) [32, 33]. For example, some studies indicate that long-term exposure to pollutants such as PM2.5 and NO_2_ increases cancer risk, but there is limited empirical research on the impact of policy interventions on cancer outcomes by improving environmental quality [34, 35]. Our study further demonstrated that policy interventions to reduce pollutant emissions can effectively reduce cancer incidence, providing direct evidence of the health benefits of such policies. While lung cancer is most directly linked to air pollution, the policy’s effect on hormone-related cancers (e.g., breast, endometrial) appears stronger, possibly reflecting broader socioeconomic and psychosocial influences under CETP. Additionally, the policy’s impact is more pronounced in highly polluted regions and among individuals with poorer mental health. These findings highlight that a comprehensive environmental policy such as CETP can deliver multifaceted public health benefits, underscoring the need for integrated strategies that address both environmental and psychosocial determinants of cancer.

Compared to existing research on carbon reduction policies in other countries [36, 37], our study provides a more detailed exploration of the differential impact of CETP on cancer risk among Chinese middle-aged and elderly populations, considering gender, BMI, and smoking status. Although studies have shown differences in cancer susceptibility between males and females [38], our study further revealed that CETP had a more pronounced effect in reducing cancer incidence among females, providing new evidence on the differential effects of environmental policies by gender. Additionally, the subgroup analysis showed that individuals with higher BMI and smokers had weaker responses to the policy, consistent with findings that obesity and smoking increase cancer risk, suggesting that these lifestyle factors may affect the effectiveness of policy interventions. Our study also has certain limitations. Our data were primarily from specific regions, and the generalizability of the results may be limited. More validation studies are needed across different regions and populations. Lastly, the latency period for cancer development is long, and the study’s time window may not fully capture the long-term effects of the policy.

## Conclusion

CETP significantly reduced the risk of several cancers, such as breast, cervical, and gastric cancers, with particularly strong effects in highly polluted environments and among individuals with poorer mental health. By using a staggered DID model, parallel trend test, entropy balancing, and subgroup analyses, the robustness and reliability of the findings were validated. These findings suggest that environmental policies have an important positive role in public health, not only reducing the adverse health effects of environmental pollution but also improving health outcomes for vulnerable populations. Despite certain limitations, this study provides empirical support for the effectiveness of environmental policies as a public health intervention. Future studies should further investigate the long-term effects of CETP and verify its applicability globally under different contexts to confirm its broader significance in cancer prevention.

## Supplementary information

**Additional file 1:** **Figure S1:** Flowchart of Participant Inclusion and Exclusion.

**Additional file 2:** **Figure S2:** Parallel Trend Test for Cancer Incidence Before and After Policy Implementation.

**Additional file 3:** **Figure S3:** Placebo Test on Pan-cancer Model.

**Additional file 4:** **Figure S4:** Heatmap of the Association Between Common Air Pollutants and Cancer Types.

**Additional file 5:** **Table S1:** Dynamic Effects of CETP on Pan-Cancer Incidence.

**Additional file 6:** **Table S2:** Impact of CETP on Cancer Incidence After Entropy Balancing and PSM.

**Additional file 7:** **Table S3:** Impact of CETP on Pan-cancer Incidence Using BMI-Imputed Dataset.

**Additional file 8:** **Table S4:** Impact of CETP on Pan-Cancer Incidence with Re-inclusion of New Migrant Participants.

**Additional file 9:** **Table S5:** Impact of CETP on Pan-Cancer Incidence with Extended Policy and Residence Control.

## Declarations

### Fundings

The study was supported by the educational research project of Nanjing Medical University (2023YJS-LX011 and 2023YJS-LX012), National Natural Science Foundation of China (82102073 and 82203216), and Natural Science Foundation of Jiangsu Province (BK20220724).

### Availability of data and materials

Data available on request.
