# Supplementary figures and images for: Evaluating the impact of Carbon Emission Trading Policy on pan-cancer incidence among middle-aged and elderly populations: a quasi-natural experiment
Source: Environ Health Prev Med. 2025 May 29;30:43. doi: 10.1265/ehpm.24-00387 (PMC12127080; doi:10.1265/ehpm.24-00387)

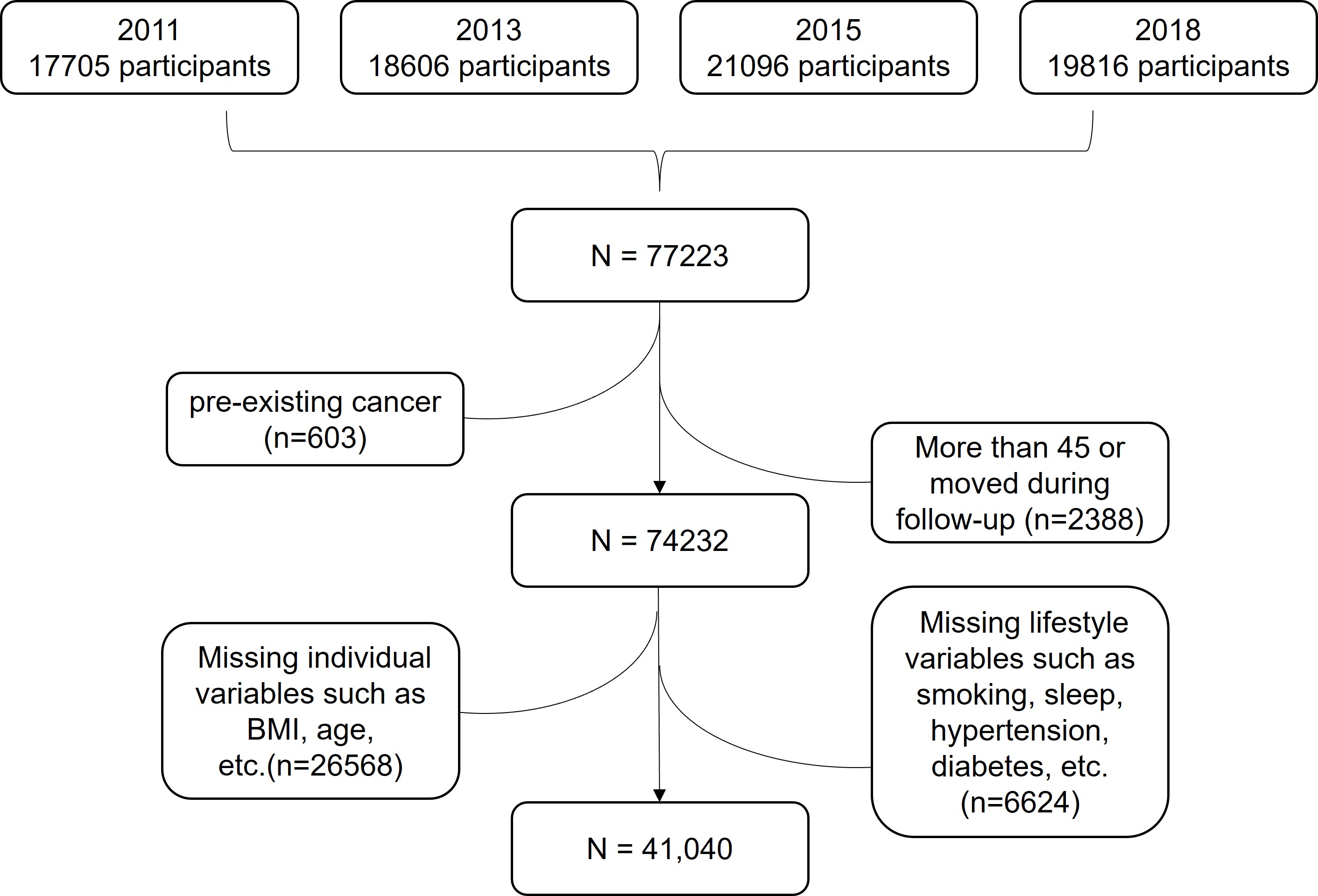

Supplement: Supplementary file 1 — Additional file 1: Figure S1: Flowchart of Participant Inclusion and Exclusion. [file ehpm-30-043-s001.jpg]

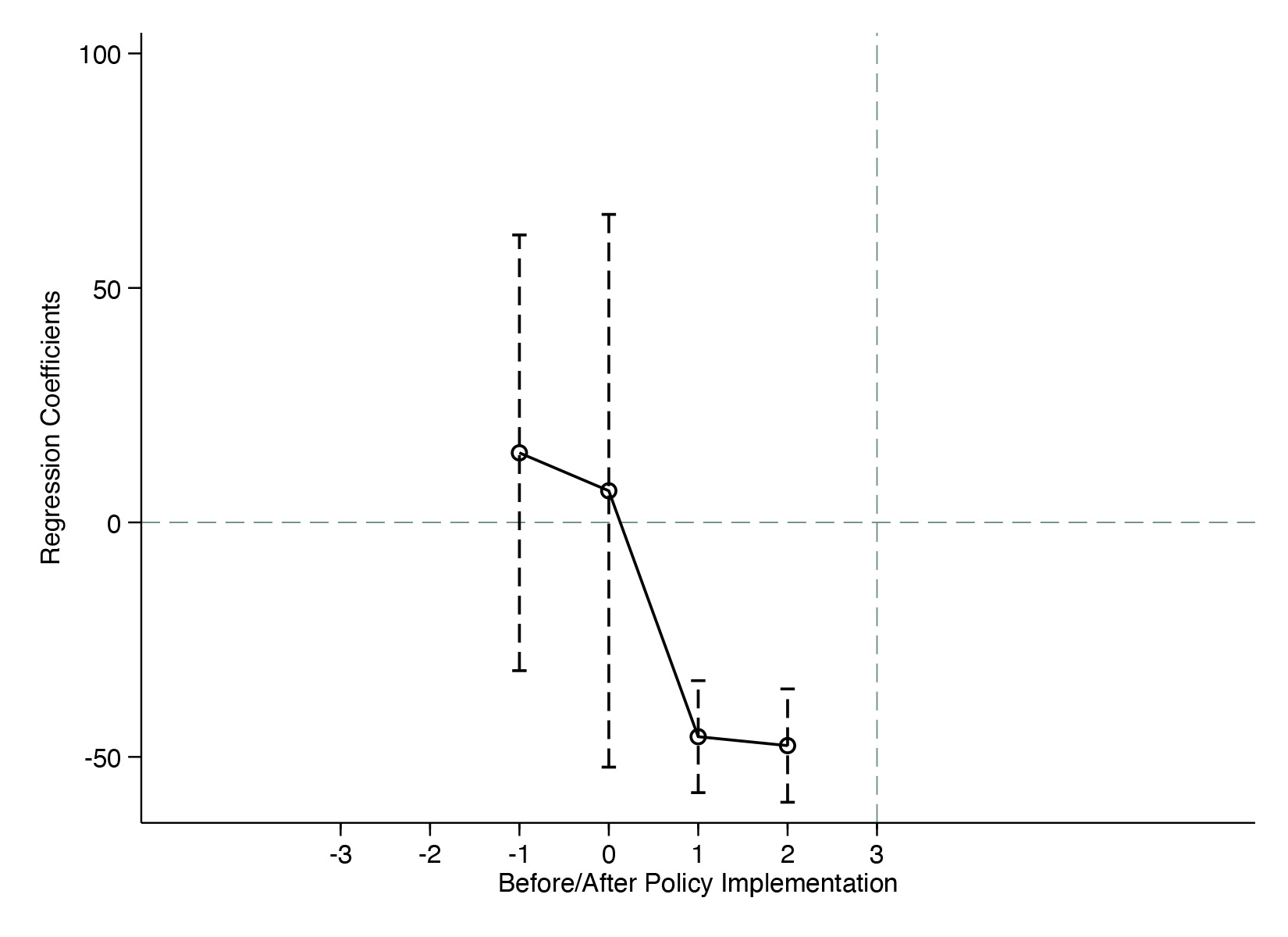

Supplement: Supplementary file 2 — Additional file 2: Figure S2: Parallel Trend Test for Cancer Incidence Before and After Policy Implementation. [file ehpm-30-043-s002.jpg]

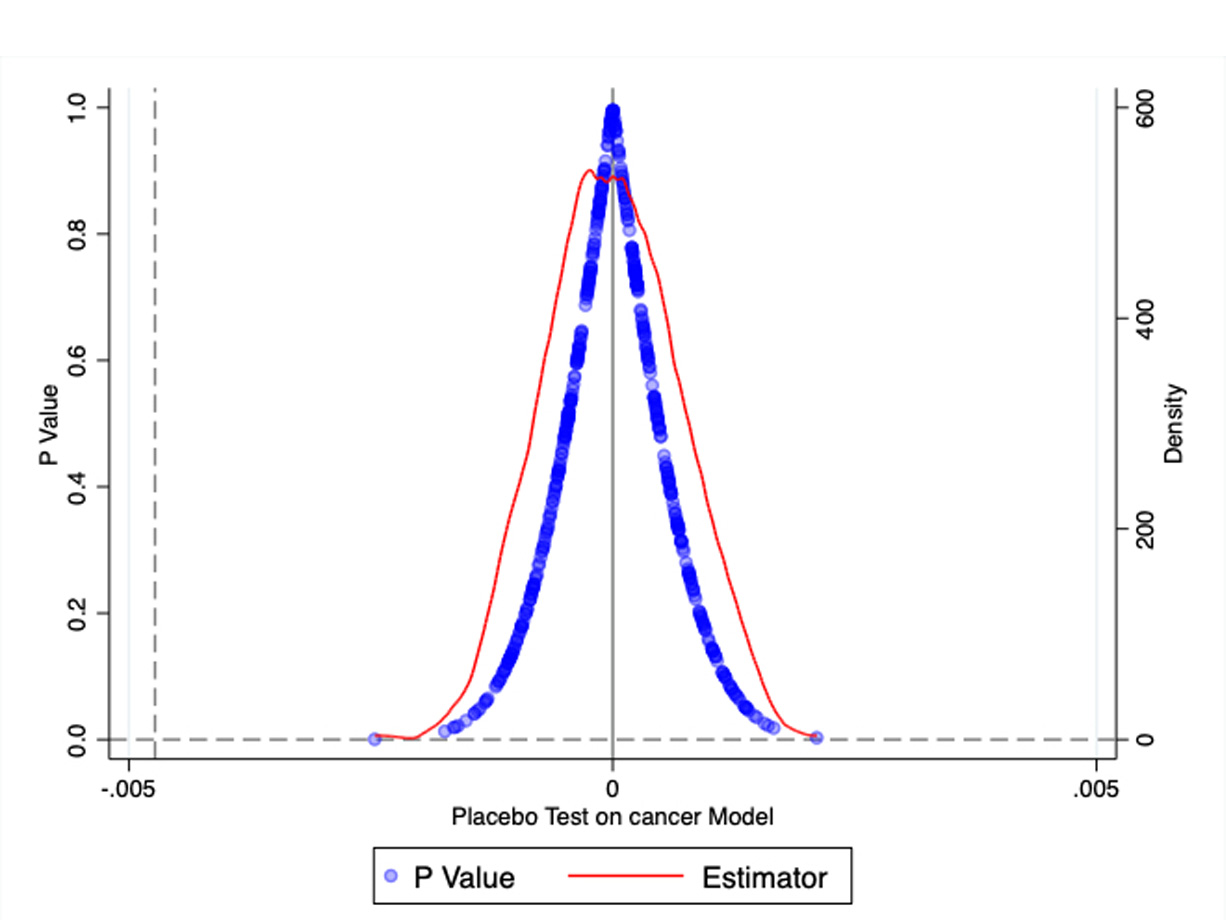

Supplement: Supplementary file 3 — Additional file 3: Figure S3: Placebo Test on Pan-cancer Model. [file ehpm-30-043-s003.jpg]

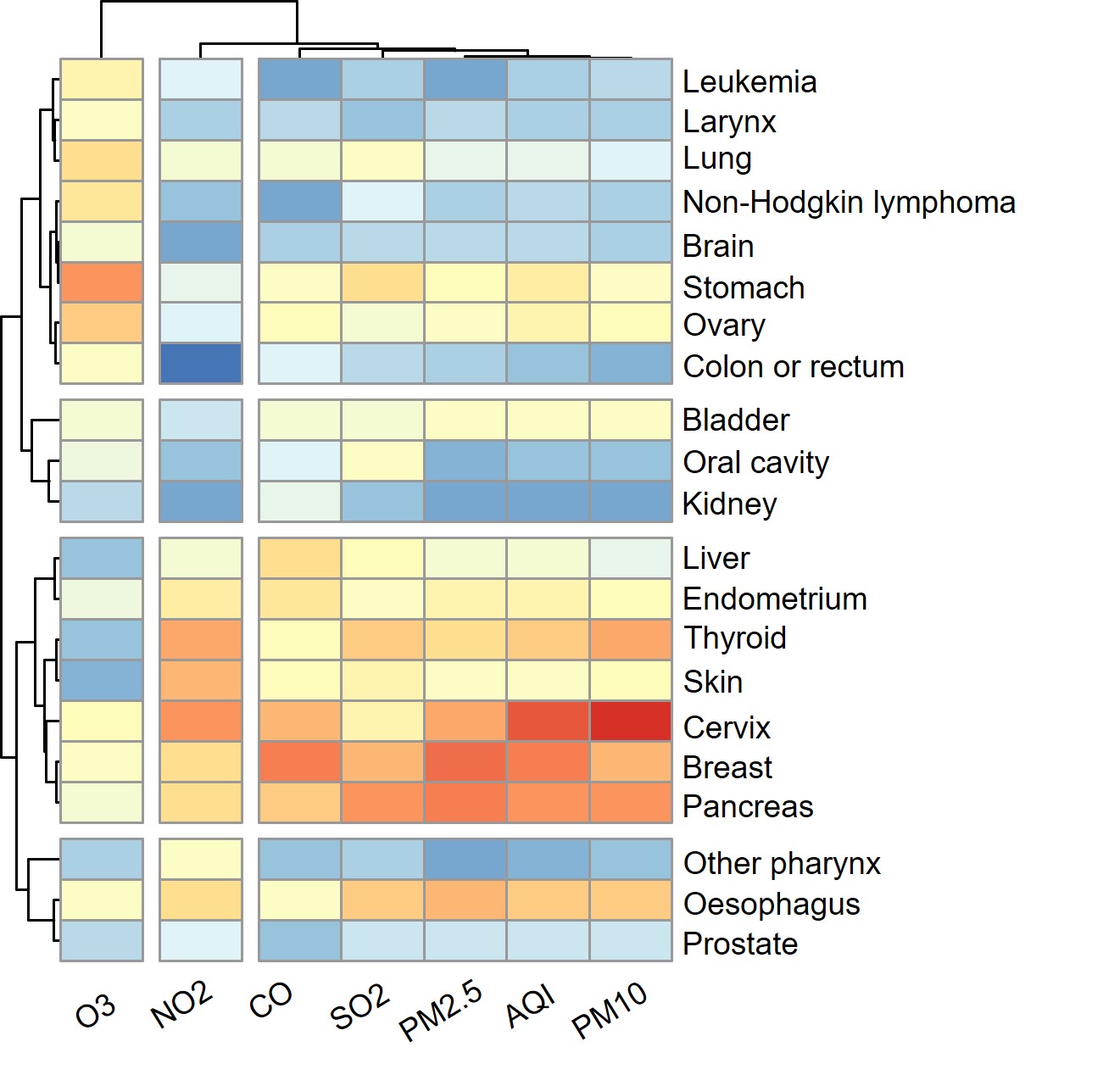

Supplement: Supplementary file 4 — Additional file 4: Figure S4: Heatmap of the Association Between Common Air Pollutants and Cancer Types. [file ehpm-30-043-s004.jpg]
