# Supplementary material for: Evaluating the impact of Carbon Emission Trading Policy on pan-cancer incidence among middle-aged and elderly populations: a quasi-natural experiment
Source: Environ Health Prev Med. 2025 May 29;30:43. doi: 10.1265/ehpm.24-00387 (PMC12127080; doi:10.1265/ehpm.24-00387)
Supplement: Supplementary file 5 — Additional file 5: Table S1: Dynamic Effects of CETP on Pan-Cancer Incidence. [file ehpm-30-043-s005.docx]

| Variables* | Model 1 | p | Model 2 | p |
| --- | --- | --- | --- | --- |
| d_1 | -14.030  [-41.672, 13.613] | 0.320 | -8.802  [-45.189, 27.585] | 0.635 |
| d0 | -2.224  [-43.339, 38.890] | 0.916 | 2.858  [-54.536, 60.253] | 0.922 |
| d1 | -36.353  [-46.525, -26.181] | <0.001 | -33.727  [-44.991, -22.463] | <0.001 |
| d2 | -37.554  [-47.790, -27.319] | <0.001 | -35.503  [-46.716, -24.289] | <0.001 |
| _cons | 78.911  [42.807, 115.015] | <0.001 | 97.220  [48.103, 146.337] | <0.001 |
| R² | 0.0014 |  | 0.0017 |  |
| N | 40,895 |  | 34,264 |  |

Table S1: Dynamic Effects of CETP on Pan-Cancer Incidence^#^

^#^Model 1 controls for core variables including gender, age, and BMI. Model 2 further incorporates additional covariates, such as education, rural residency, sleep duration, smoking status, alcohol consumption, hypertension history, and diabetes history.

* d_1 indicates the period one year before the event (t = -1), d0 represents the event year (t = 0), and d1 and d2 correspond to the first (t = +1) and second (t = +2) years after the event, respectively.
