# Supplementary material for: Evaluating the impact of Carbon Emission Trading Policy on pan-cancer incidence among middle-aged and elderly populations: a quasi-natural experiment
Source: Environ Health Prev Med. 2025 May 29;30:43. doi: 10.1265/ehpm.24-00387 (PMC12127080; doi:10.1265/ehpm.24-00387)
Supplement: Supplementary file 6 — Additional file 6: Table S2: Impact of CETP on Cancer Incidence After Entropy Balancing and PSM. [file ehpm-30-043-s006.docx]

Table S2: Impact of CETP on Cancer Incidence After Entropy Balancing and PSM

|  | Entropy Balancing | | PSM | |
| --- | --- | --- | --- | --- |
| CETP | coef [95% CI] | p | coef [95% CI] | p |
| Lung | -5.698  [-10.616, -0.781] | 0.023 | -4.220  [-8.412, -0.027] | 0.049 |
| Breast | -10.172  [-15.951, -4.393] | 0.001 | -9.083  [-15.374, -2.794] | 0.005 |
| Oesophagus | -6.182  [-11.249, -1.114] | 0.017 | -6.064  [-11.039, -1.089] | 0.017 |
| Stomach | -6.392  [-12.018, -0.766] | 0.026 | -4.813  [-9.287, -0.339] | 0.035 |
| Cervix | -9.024  [-14.292, -3.756] | 0.001 | -8.810  [-14.927, -2.692] | 0.005 |
| Endometrium | -9.335  [-14.781, -3.888] | 0.001 | -10.199  [-16.910, -3.488] | 0.003 |
