# Supplementary material for: Evaluating the impact of Carbon Emission Trading Policy on pan-cancer incidence among middle-aged and elderly populations: a quasi-natural experiment
Source: Environ Health Prev Med. 2025 May 29;30:43. doi: 10.1265/ehpm.24-00387 (PMC12127080; doi:10.1265/ehpm.24-00387)
Supplement: Supplementary file 7 — Additional file 7: Table S3: Impact of CETP on Pan-cancer Incidence Using BMI-Imputed Dataset. [file ehpm-30-043-s007.docx]

| Variables | Model 1  Coef [95% CI] | p | Model 2  Coef [95% CI] | p |
| --- | --- | --- | --- | --- |
| CETP × POST | -18.250  [-31.767, -4.733] | 0.008 | -15.250  [-29.624, -0.876] | 0.038 |
| Gender | -26.721  [-34.677, -18.765] | <0.001 | -14.316  [-26.315, -2.317] | 0.019 |
| Age | -0.635  [-1.068, -0.202] | 0.004 | -0.673  [-1.172, -0.175] | 0.008 |
| BMI | -0.0006  [-0.0011, -0.00004] | 0.036 | -0.019  [-0.046, 0.008] | 0.170 |
| Education |  |  | 3.668  [-1.106, 8.443] | 0.132 |
| Rural |  |  | -6.666  [-16.311, 2.978] | 0.175 |
| Sleep |  |  | -2.252  [-4.785, 0.281] | 0.081 |
| Smoke |  |  | -22.776  [-31.951, -13.601] | <0.001 |
| Drink |  |  | -3.189  [-13.478, 7.100] | 0.544 |
| Hypertension |  |  | 3.649  [-5.323, 12.621] | 0.425 |
| Diabetes |  |  | 5.804  [-8.195, 19.803] | 0.416 |
| _cons | 83.433  [53.850, 113.016] | <0.001 | 96.247  [56.258, 136.236] | <0.001 |
| r2 | 0.0025 |  | 0.0030 |  |
| N | 67,754 |  | 61,078 |  |

Table S3: Impact of CETP on Pan-cancer Incidence Using BMI-Imputed Dataset^#^

^#^Model 1 controls for core variables including gender, age, and BMI. Model 2 further incorporates additional covariates, such as education, rural residency, sleep duration, smoking status, alcohol consumption, hypertension history, and diabetes history.
