# Supplementary material for: Evaluating the impact of Carbon Emission Trading Policy on pan-cancer incidence among middle-aged and elderly populations: a quasi-natural experiment
Source: Environ Health Prev Med. 2025 May 29;30:43. doi: 10.1265/ehpm.24-00387 (PMC12127080; doi:10.1265/ehpm.24-00387)
Supplement: Supplementary file 8 — Additional file 8: Table S4: Impact of CETP on Pan-Cancer Incidence with Re-inclusion of New Migrant Participants. [file ehpm-30-043-s008.docx]

| Variables | Model 1  Coef [95% CI] | p | Model 2  Coef [95% CI] | p |
| --- | --- | --- | --- | --- |
| CETP × POST | -19.463  [-34.997, -3.929] | 0.014 | -17.100  [-33.589, -0.610] | 0.042 |
| Gender | -33.900  [-43.373, -24.426] | <0.001 | -7.160  [-21.832, 7.513] | 0.339 |
| Age | -0.802  [-1.286, -0.317] | 0.001 | -1.017  [-1.574, -0.459] | <0.001 |
| BMI | -0.001  [-0.002, -0.000] | 0.027 | -0.031  [-0.062, 0.001] | 0.056 |
| Education |  |  | 1.515  [-4.449, 7.479] | 0.619 |
| Rural |  |  | -12.132  [-23.992, -0.273] | 0.045 |
| Sleep |  |  | -3.675  [-6.555, -0.795] | 0.012 |
| Smoke |  |  | -39.299  [-50.362, -28.237] | <0.001 |
| Drink |  |  | -15.561  [-27.101, -4.022] | 0.008 |
| Hypertension |  |  | -2.616  [-13.140, 7.908] | 0.626 |
| Diabetes |  |  | 10.922  [-5.623, 27.466] | 0.196 |
| _cons | 102.739  [29.854,175.625] | 0.006 | 154.740  [107.311, 202.170] | <0.001 |
| r2 | 0.002 |  | 0.003 |  |
| N | 68,441 |  | 61,716 |  |

Table S4: Impact of CETP on Pan-Cancer Incidence with Re-inclusion of New Migrant Participants^#^

^#^Model 1 controls for core variables including gender, age, and BMI. Model 2 further incorporates additional covariates, such as education, rural residency, sleep duration, smoking status, alcohol consumption, hypertension history, and diabetes history.
