# Supplementary material for: Evaluating the impact of Carbon Emission Trading Policy on pan-cancer incidence among middle-aged and elderly populations: a quasi-natural experiment
Source: Environ Health Prev Med. 2025 May 29;30:43. doi: 10.1265/ehpm.24-00387 (PMC12127080; doi:10.1265/ehpm.24-00387)
Supplement: Supplementary file 9 — Additional file 9: Table S5: Impact of CETP on Pan-Cancer Incidence with Extended Policy and Residence Control. [file ehpm-30-043-s009.docx]

| Variables | Model 1 | p | Model 2 | p | Model 3 | p | Model 4 | p |
| --- | --- | --- | --- | --- | --- | --- | --- | --- |
| CETP × POST | -38.563  [-53.738, -23.389] | <0.001 | -37.617  [-49.225, -26.008] | <0.001 | -34.846  [-45.796, -23.896] | <0.001 | -15.358  [-29.737，-0.979] | 0.036 |
| Low-carbon | 5.642 |  |  |  |  |  |  |  |
| Energy-Use | [45.432, 144.529] | <0.001 | -12.290  [-24.294, -0.286] | <0.001 |  |  |  |  |
| Duration of Residence |  |  |  |  | -5.893  [-15.960, 4.174] | 0.251 | -5.308  [-10.281, 2.537] | 0.036 |
| Gender | -21.802  [-35.378, -8.225] | 0.002 | -21.259  [-34.757, -7.761] | 0.002 | -22.102  [-35.676, -8.528] | 0.001 | -14.886  [-26.941, 6.150] | 0.016 |
| Age | -0.609  [-1.280, 0.062] | 0.075 | -0.613  [-1.284, 0.058] | 0.073 | -0.516  [-1.217, 0.186] | 0.149 | -0.473  [-1.770, 0.267] | 0.076 |
| Education | 3.111  [-2.857, 9.078] | 0.307 | 2.802  [-3.193, 8.797] | 0.360 | 3.341  [-2.617, 9.300] | 0.272 | 4.122  [-0.657, 8.902] | 0.091 |
| BMI | -0.0118  [-0.0477, 0.0241] | 0.519 | -0.0127  [-0.0489, 0.0235] | 0.491 | -0.0117  [-0.0479, 0.0244] | 0.524 | -0.017  [-1.240, 0.215] | 0.215 |
| Rural | -7.219  [-19.190, 4.752] | 0.237 | -6.905  [-18.843, 5.034] | 0.257 | -6.621  [-18.592, 5.350] | 0.278 | -5.392  [-15.042, 4.924] | 0.273 |
| Sleep | -3.310  [-6.243, -0.377] | 0.027 | -3.381  [-6.307, -0.455] | 0.024 | -3.354  [-6.285, -0.424] | 0.025 | -2.289  [-4.820, 1.291] | 0.076 |
| Smoke | -16.929  [-26.294, -7.563] | <0.001 | -17.446  [-26.969, -7.923] | <0.001 | -16.750  [-26.181, -7.318] | <0.001 | -22.718  [-31.892, 4.681] | <0.001 |
| Drink | -0.521  [-13.676, 12.633] | 0.938 | -0.556  [-13.676, 12.564] | 0.934 | -0.733  [-13.850, 12.383] | 0.913 | -3.314  [-13.596, 6.969] | 0.528 |
| Hypertension | 4.819  [-6.626, 16.264] | 0.409 | 4.372  [-7.116, 15.860] | 0.456 | 4.768  [-6.675, 16.210] | 0.414 | 3.647  [-5.324, 12.618] | 0.426 |
| Diabetes | 3.726  [-14.370, 21.822] | 0.687 | 3.829  [-14.258, 21.916] | 0.678 | 3.412  [-14.768, 21.592] | 0.713 | 5.857  [-8.134, 19.849] | 0.412 |
| _cons | 94.981  [45.432, 144.529] | <0.001 | 100.906  [51.277, 150.534] | <0.001 | 100.972  [51.029, 150.916] | <0.001 | 104.551  [4.960, 145.842] | <0.001 |
| R² | 0.0017 |  | 0.0018 |  | 0.0018 |  | 0.0031 |  |
| N | 34,264 |  | 34,264 |  | 34,264 |  | 61,078 |  |

Table S5: Impact of CETP on Pan-Cancer Incidence with Extended Policy and Residence Controls^#^

^#^ All Four models incorporate controls for gender, age, BMI, education level, rural residency, sleep duration, smoking status, alcohol consumption, hypertension history, and diabetes history. Model 1 additionally includes a control variable for the Low-Carbon City Policy, Model 2 adds a control variable for the Energy Use Rights Pilot Policy, Model 3 further includes a control for the duration of individual residence (excluding new in-migrants) and Model 4 includes a control for the duration of individual residence (including new in-migrants).
